# Integrating appreciative education with AI-assisted oral training for sustainable EFL learning: a study on speaking anxiety and oral proficiency

**DOI:** 10.3389/fpsyg.2026.1803848

**Published:** 2026-04-10

**Authors:** Fangli Zhang, Meng Yang

**Affiliations:** 1 Luoyang Normal University, Luoyang, China; 2 Shaanxi Institute of International Trade and Commerce, Xi'an, China; 3 UCSI University, Cheras, Malaysia

**Keywords:** AI-assisted oral training, appreciative education, blended learning, Foreign language speaking anxiety, oral proficiency, SDG4, sustainable EFL learning

## Abstract

**Background:**

Foreign language speaking anxiety constitutes a persistent barrier to oral proficiency development among Chinese college students; however, prevailing technology-centered instructional approaches inadequately address learners' affective needs, thereby limiting their effectiveness in alleviating anxiety and fostering intrinsic motivation. This study proposed a blended pedagogical model—the “Praise-Speak” program that integrates offline appreciative education with AI-assisted oral training via the FIF platform.

**Methods:**

A two-phase mixed-methods design was employed. In the first phase, large-scale surveys (*n* = 1,044 for anxiety assessment; *n* = 209 for appreciative education needs) identified freshmen as the priority intervention cohort. In the second phase, a 16-week pilot intervention was conducted with 13 participants, utilizing a pretest-posttest design supplemented by reflective journal analysis.

**Results:**

The findings demonstrated statistically significant improvements in IELTS speaking scores (*p* = 0.005) and a marked reduction in speaking anxiety (*p* < 0.001). Qualitative analysis revealed three core mechanisms underlying these outcomes: the establishment of psychological safety, positive receptivity to appreciative pedagogy, and technology-humanism synergy.

**Conclusion:**

This study offers a replicable pedagogical framework that harmonizes technological empowerment with humanistic care, contributing to the realization of the United Nations Sustainable Development Goal 4.

## 1 Introduction

In the context of global educational reform, the United Nations Sustainable Development Goal 4 (SDG 4) emphasizes the importance of ensuring inclusive and equitable quality education while promoting lifelong learning opportunities for all (United Nations, 2015; Smaniotto et al., 2023). Within this framework, addressing the affective dimensions of language learning has emerged as a critical concern, as learners' psychological well-being directly influences their capacity for sustained engagement and long-term academic success (Dincer and Yesilyurt, 2017; Ornelas et al., 2025). In the domain of English as a Foreign Language (EFL) education, Chinese college students commonly encounter the challenge of foreign language speaking anxiety (FLSA), which constitutes a persistent barrier to oral proficiency development and undermines intrinsic learning motivation (Liu, 2018; Dana and Aminatun, 2022; Sari, 2023).

While technology-enhanced learning platforms such as MOOCs, SPOCs, learning management systems, and AI-assisted oral training tools including the FIF Speaking English Platform (hereinafter referred to as FIF platform) have demonstrated considerable potential in delivering language practice and providing timely feedback (Ebadi et al., 2025), purely technology-centered approaches present inherent limitations. Specifically, the absence of humanistic interaction in such systems often fails to address learners' social and emotional needs, thereby reducing their effectiveness in mitigating speaking anxiety (Zhang and Liu, 2013; Abdelhalim and Alsalem, 2019). Conversely, appreciative education (AE), a positive and affirming pedagogical approach rooted in strength-based philosophy (Whitney and Cooperrider, 2011; Bloom et al., 2013), has been recognized as an effective means of fulfilling students' fundamental psychological needs for competence and relatedness (Deci and Ryan, 2012; Hilao-Valencia and Ortega-Dela Cruz, 2023). However, limited empirical research has examined the integrated application of appreciative education with technology-enhanced platforms in EFL contexts, particularly regarding their synergistic effects on speaking anxiety reduction and motivation enhancement.

To address this gap, the present study proposes a blended pedagogical model—the “Praise-Speak” program that integrates offline appreciative education with AI-assisted oral training via the FIF platform. Through a two-phase mixed-methods design (Creswell and Clark, 2017), this research empirically investigates the model's effectiveness in reducing speaking anxiety and enhancing oral proficiency among Chinese university freshmen. This study contributes to the field by offering a replicable framework that harmonizes technological empowerment with humanistic care, thereby advancing sustainable and inclusive EFL education in alignment with SDG 4.

## 2 Literature review

### 2.1 Heterogeneity of foreign language speaking anxiety

A substantial body of research has confirmed that foreign language speaking anxiety exhibits significant variation across EFL of different school ages and ability levels. Recognizing these differences has become a central focus in intervention-related studies. For example, several studies have demonstrated a significant negative correlation between anxiety levels and oral performance (Dana and Aminatun, 2022; Sari, 2023).

Additional findings indicate that the impact of anxiety is moderated by individual differences. Notably, a large-scale stratified analysis of Chinese students revealed that lower-proficiency learners are more adversely affected by anxiety, although the effective application of coping strategies can reduce its negative impact by approximately 15% (Liu, 2018). These findings suggest that the sources and severity of anxiety may differ meaningfully across school age and proficiency groups. Consequently, assessing anxiety levels within distinct student populations and identifying those most in need of support is essential for the development of targeted and effective intervention strategies.

### 2.2 Technology enhanced learning lacks emotional support

Numerous educators have adopted technology enhanced learning platforms, such as FIF, MOOCs, and SPOCs to deliver language practice and timely feedback, demonstrating considerable potential in enhancing students' language proficiency. However, purely technology driven approaches present inherent limitations. Due to the absence of human interaction in artificial intelligence systems, these platforms often fail to address learners' social and emotional needs. Specifically, within skill focused training activities on digital platforms, the lack of humanistic care and pedagogical empathy from instructors may result in a diminished sense of belonging among students. This emotional gap can reduce the effectiveness of technological tools in mitigating speaking communication anxiety. Empirical evidence suggests that students may have limited responses to intervention measures that mainly rely on technological means or traditional teaching methods, especially in addressing the emotional aspects of language learning (Zhang and Liu, 2013).

In contrast, emerging research suggests that integrating technology with appreciative education into classroom teaching enables students to more effectively identify weaknesses in their speaking performance (Abdelhalim and Alsalem, 2019). Recently, scholars have begun to study the role of learners' emotions and psychological mechanisms in the artificial intelligence-assisted English as a foreign language teaching environment. For instance, Fan and Zhang (2024) found that in an AI-enhanced EFL teaching environment, AI literacy can indirectly promote learners' willingness to communicate in a foreign language by enhancing their AI learning self-efficacy and reducing foreign language classroom anxiety. Additionally, Zhang et al. (2025) pointed out that AI literacy can also indirectly influence learners' intention to engage in AI-supported learning behaviors through their attitudes toward AI-assisted learning and their pleasant experiences in foreign language learning. These studies collectively emphasize the importance of paying attention to learners' psychological and emotional factors in AI-enabled foreign language teaching. Collectively, these findings underscore the insufficiency of purely technological interventions in delivering adequate emotional support (Ma et al., 2025; Shi, 2026; Shuai, 2025). Thus, it has established the theoretical necessity and practical feasibility of integrating AE with technology-enhanced language learning in a blended teaching model.

### 2.3 Appreciative education is an effective way to provide emotional support

Appreciative Education (AE) from the perspective of pedagogical methodology (Bloom et al., 2013), it represents a positive, affirming, and discovery oriented approach that effectively addresses the emotional deficiencies inherent in technology-driven learning environments. Some scholars believe that AE is crucial for higher education (Mather et al., 2025). The successful implementation of this approach hinges on the identification and acknowledgment of students' strengths (Whitney and Cooperrider, 2011), which contributes to the cultivation of a supportive and conducive classroom atmosphere. Such teacher-facilitated environments are widely recognized as critical in enhancing students' intrinsic motivation to learn (Dincer and Yesilyurt, 2017). And Lestari (2018) also found that the lack of affirming and supportive interactions in the learning context is an important factor contributing to students' underperformance.

From a psychological standpoint, appreciative education effectively fulfills students' fundamental psychological needs, particularly those related to competence and belongingness (Deci and Ryan, 2012). Empirical evidence from Hilao-Valencia and Ortega-Dela Cruz (2023) demonstrates that AE not only increases the frequency with which students employ effective learning strategies but also exerts a notably significant effect in reducing anxiety levels among learners with lower proficiency. Consistent with this view, existing EFL research indicates that teachers' affective teaching behaviors (such as encouragement and emotional support) can significantly enhance learners' perseverance, enjoyment of learning, and their sustained engagement in language learning (Hu et al., 2022; Derakhshan et al., 2025). As such, it serves as a valuable intervention for rebuilding student confidence and enhancing oral communication abilities. When systematically integrated into classroom practice, teacher recognition and verbal encouragement foster a psychologically safe environment, enabling students to experience a sense of achievement in English language learning. These repeated positive experiences reinforce intrinsic motivation through the accumulation of success. Consequently, incorporating the emotional support mechanisms of AE into technology-enhanced learning environments offers a promising strategy for addressing the underlying psychological causes of oral language anxiety.

### 2.4 Theoretical integration: building a synergistic framework

This study draws upon Vygotsky's Zone of Proximal Development (ZPD) theory and Self-Determination Theory (SDT) to examine the potential effectiveness of integrating AE with the FIF platform (Vygotsky, 1978; Deci and Ryan, 2012). A conceptual framework is proposed in which ZPD delineates the cognitive space within which learning takes place, while SDT provides the motivational foundation that drives engagement within this space. The technological platform (FIF) fosters learners' sense of competence through structured feedback mechanisms, whereas teachers' implementation of appreciative education supports students' need for relatedness through empathetic and humanistic interaction. These complementary processes function synergistically to enhance English language instruction.

#### 2.4.1 Zone of proximal development theory

This study is grounded in Vygotsky's theory of the “Zone of Proximal Development” (ZPD), which is defined as “the distance between the actual developmental level, at which a learner can solve problems independently, and the potential developmental level that can be attained through guidance from adults or collaboration with more capable peers (Vygotsky, 1978; Ma et al., 2025; Shuai, 2025). Effective instruction should take place within this zone. In the present research, the FIF platform functions as a supportive tool by delivering immediate and objective feedback to enhance students' language proficiency.

#### 2.4.2 Self-determination theory

Self-Determination Theory (SDT) was a well-established theoretical framework in psychology, was developed by Richard M. Ryan and Edward L. Deci during the early 1970s. According to SDT, students' motivation for learning is determined by three fundamental psychological needs: competence, relatedness, and autonomy, which in turn influence academic achievement (Deci and Ryan, 2012; Ma et al., 2025; Shi, 2026). The FIF platform functions as a virtual instructor, supporting students within their ZPD by enhancing competence through structured repetitive practice and timely feedback. Concurrently, teachers' appreciative and supportive pedagogy fosters a secure and nurturing learning environment, offering emotional scaffolding that addresses students' need for relatedness.

**Figure 1:**
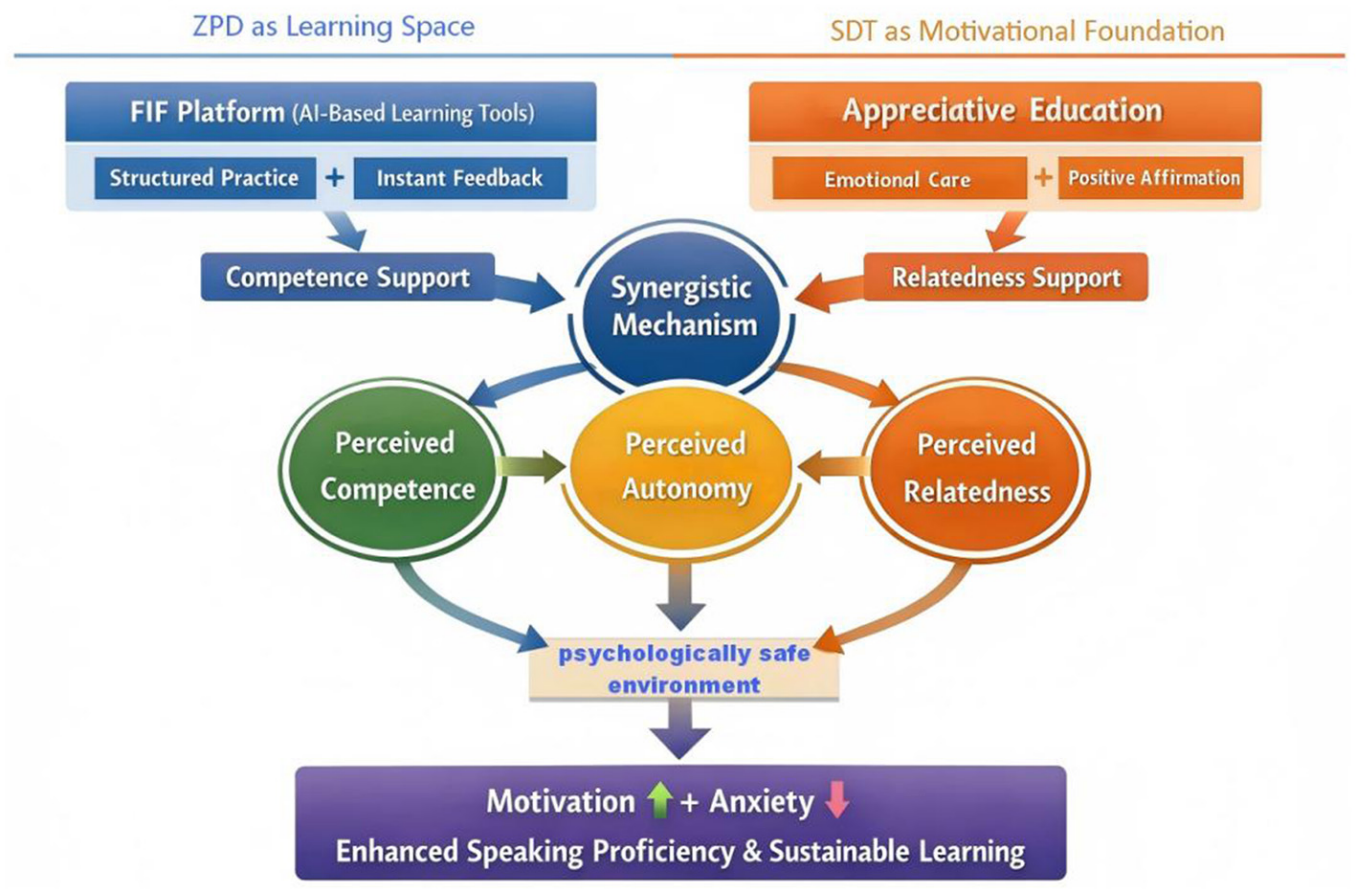
An integrated conceptual framework of ZPD and SDT in AI-assisted EFL speaking learning.

As shown in Figure 1, the integrated conceptual framework proposed in this study reveals the synergistic effect of the technological and humanistic paths. The AI-assisted FIF platform, as a foreign language learning support tool, provides structured exercises and immediate feedback within the learner's ZPD, thereby enhancing the learner's perceived competence and improving skills. Meanwhile, the appreciative education implemented by teachers, through emotional encouragement and positive affirmation, directly responds to the learner's perceived relatedness, creating a psychologically safe classroom atmosphere. Under the “synergistic mechanism,” the two paths jointly promote the learner's perceived autonomy. This framework emphasizes that the parallel effect of cognitive support and emotional needs satisfaction not only enhances learning motivation but also alleviates language anxiety and promotes the development of oral language skills and continuous learning.

### 2.5 Sustainable education: an overall framework for language learning

Contemporary education places growing emphasis on sustainable learning, which entails fostering learners' capacities for lifelong learning, psychological resilience, and the perception of well-being to ensure the enduring impact of educational outcomes (Smaniotto et al., 2023; Ornelas et al., 2025). This perspective is closely aligned with the United Nations' Sustainable Development Goal 4 (SDG 4), which promotes inclusive and equitable quality education. Traditional approaches to English language instruction, as well as purely technology-driven learning models, have proven insufficient in fully supporting this objective. Empirical evidence indicates that when teachers overlook students' emotional needs, it may negatively affect their motivation and engagement in learning (Dincer and Yesilyurt, 2017). Consequently, developing a hybrid teaching model that integrates technological empowerment with humanistic support has emerged as a critical strategy for advancing sustainable language learning. Within this context, the present study situates the “Prise-Speak” program to investigate how it can enhance academic engagement by satisfying learners' needs for competence and belonging (Estrada et al., 2021; Smaniotto et al., 2023), alleviate learning-related anxiety, and thereby contribute empirical insights toward achieving SDG 4-aligned language education.

### 2.6 Research question

Building upon the literature review and theoretical framework outlined above, this study seeks to investigate the following research questions:

RQ1: To what extent do students of different age groups exhibit varying needs for appreciative education, and consequently, which student group demonstrates the most urgent need for intervention through the “Praise-Speak” program?
RQ2: Can the “Praise-Speak” program significantly enhance students' oral performance and substantially reduce their speaking anxiety?

## 3 Method

Based on the aforementioned theoretical framework and identified literature gap, this study aims to empirically examine the effectiveness of the integrated model combining appreciative education with the FIF platform. To achieve this objective, a blended teaching program entitled “Praise-Speak” was implemented.

The study was conducted in accordance with the Declaration of Helsinki and approved by the Department of Foreign Language Teaching and Research, Luoyang Normal University (approval date: 25/6/2025; approval code: None). Although the university does not have a formal Institutional Review Board (IRB) for educational research, consultation and approval were obtained from the Department of Foreign Language Teaching and Research. All participants provided written informed consent prior to their inclusion in the study, having been fully informed of the study's purpose, procedures, and measures for privacy protection (all data were anonymized and securely stored). The study was conducted in full compliance with the ethical principles outlined in the Declaration of Helsinki, as well as the relevant research regulations of [China/Luoyang]. All research activities adhered to internationally recognized standards of scientific ethics.

### 3.1 Research design

**Figure 2:**
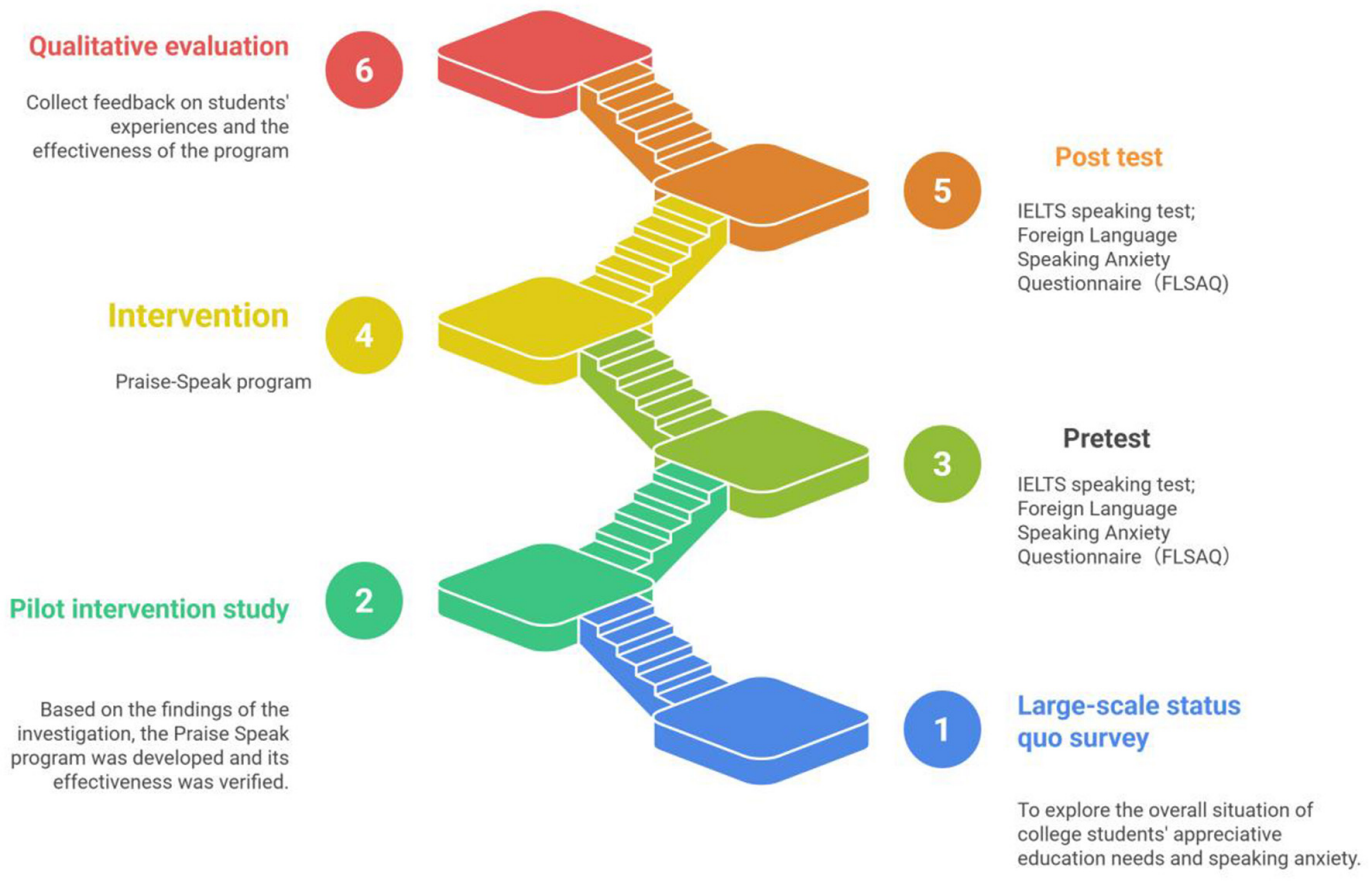
Flowchart of the research design suitable for the study.

This study employed a two-stage sequential explanatory mixed-methods design (Creswell and Clark, 2017) to systematically address the research questions. The overall research process is outlined in Figure 2.

First, during the investigation phase, the researcher employed a combination of convenience sampling and stratified sampling to conduct a comprehensive survey on foreign language speaking anxiety among English major students across all academic years at the institution where the study was conducted (*n* = 1,044). Subsequently, to assess students' needs regarding appreciative education, a proportional subsample of 209 students was selected from each grade for a supplementary questionnaire survey. This phase was designed to diagnose the current status of speaking anxiety and to accurately identify target groups for intervention.

The second stage constituted the intervention phase, during which purposive sampling was employed to select 13 willing participants from the target group identified in the initial stage. A feasibility pilot of nearly 16 weeks implementation of the “Praise-Speak” program was conducted. This phase followed a pre-test–post-test single-group design and incorporated reflective logs as a source of qualitative data. The primary objective was to assess the feasibility and effectiveness of the integrated teaching model in enhancing oral performance and alleviating anxiety through multidimensional data analysis, to guide future large-scale trials rather than establish causality (Eldridge et al., 2016; Creswell and Clark, 2017).

### 3.2 Participants and sampling

**Figure 3:**
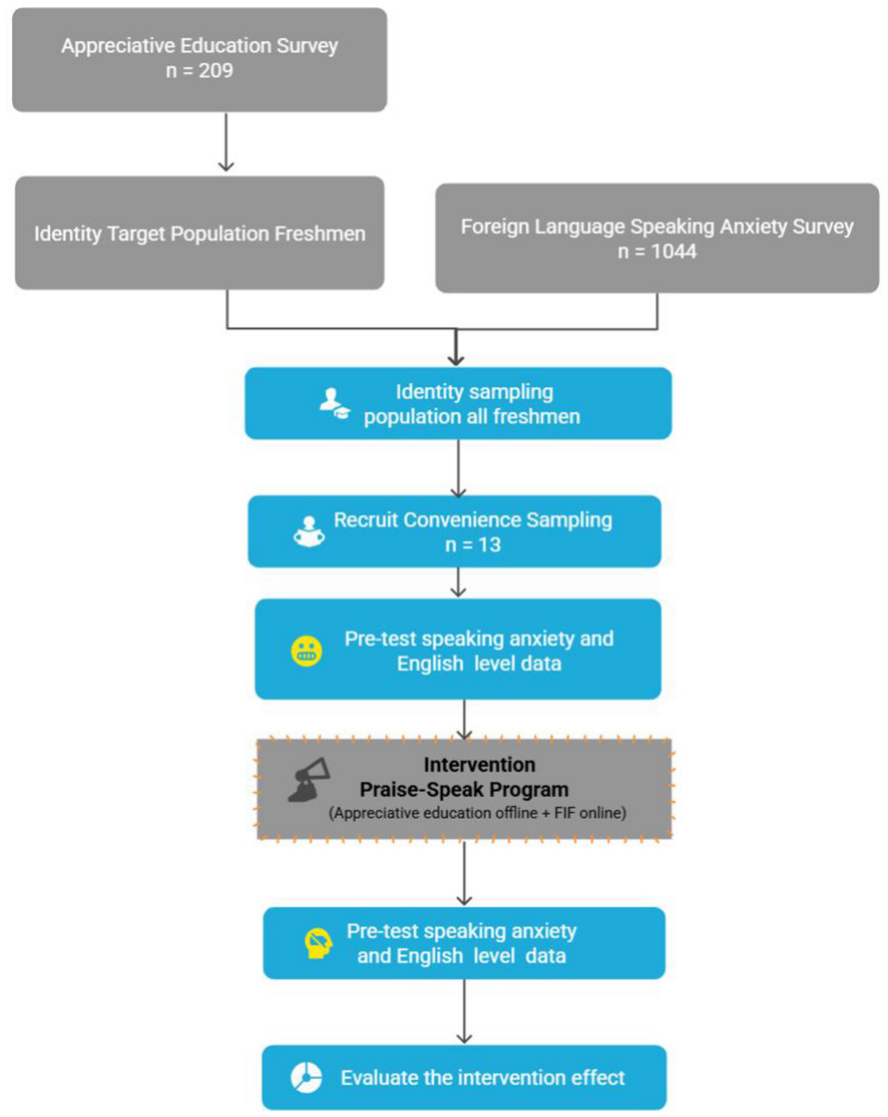
Participant selection process for the “Praise-Speak” program.

The sampling strategy employed in this study was informed by two preliminary assessments: First, a census of foreign language speaking anxiety among all English major students (*n* = 1,044) confirmed the widespread nature of the issue. Second, a survey of appreciative education needs (*n* = 209, drawn from the same population) revealed that freshmen exhibited the most pressing requirements. Consequently, freshmen were identified as the target cohort, a purposive sample of 13 participants was recruited from this population. All participants were first-year students with elevated FLSAQ scores (above the median) and volunteered to take part in the 16-week intervention. Notably, the pre-intervention anxiety levels of these participants were derived directly from their scores on the Foreign Language Speaking Anxiety Questionnaire (FLSAQ) collected during the initial phase of the census. Following the 16 weeks Praise-Speak intervention, the same instrument was administered to assess post-intervention outcomes. This approach ensured that the selected participants were both representative of the affected group and specifically relevant to the research problem. Given the exploratory nature of this pilot study aimed at assessing feasibility and generating preliminary evidence to inform future large-scale randomized controlled trials, the use of a purposive sampling design is considered appropriate and justifiable (Eldridge et al., 2016; Creswell and Clark, 2017). For the visualization flowchart of sampling, please refer to Figure 3 below.

### 3.3 Measuring tools

#### 3.3.1 Foreign language speaking anxiety questionnaire

The Foreign Language Speaking Anxiety Questionnaire (FLSAQ) employed in this study was adapted from the original scale developed by Horwitz et al. (1986), subsequently revised by Öztürk and Gürbüz (2014). The revised version comprises 33 items, of which 18 were specifically designed to assess Foreign Language Speaking Anxiety (FLSA) and were selected for use in this research. These items were measured using a five-point Likert scale to evaluate students' levels of FLSA (Horwitz et al., 1986). Psychometric analyses demonstrated strong validity and reliability within the present sample: Exploratory Factor Analysis (EFA) supported a unidimensional structure, explaining 60.82% of the total variance, with a Cronbach's alpha coefficient of 0.962 and a Kaiser–Meyer–Olkin (KMO) measure of sampling adequacy of 0.976. The complete list of items is provided in Appendix A, and the detailed EFA results are presented in Appendix E.

#### 3.3.2 Appreciative education dimension scale

The Appreciative Education Dimension Scale (AEDS) developed independently in this study, underwent a rigorous development and validation process (see Appendix B). Based on item analysis, the scale demonstrated content validity as confirmed by two experts in the fields of education and psychology. A pilot study conducted with 10 target participants enabled the identification and revision of ambiguous wording, thereby enhancing the clarity and comprehensibility of the items. Exploratory factor analysis (EFA) revealed a five-factor structure, with each dimension accounting for a cumulative variance ranging from 58.95% to 67.79%. Internal consistency, as measured by Cronbach's alpha, ranged from 0.766 to 0.850, indicating acceptable to good reliability. Kaiser–Meyer–Olkin (KMO) measures of sampling adequacy varied between 0.704 and 0.802, supporting the suitability of the data for factor analysis. The final version of the scale is presented in Appendix B, and detailed EFA results are provided in Appendix F.

#### 3.3.3 Speaking proficiency assessment

This study employs a mixed-methods assessment approach to comprehensively capture changes in students' oral language proficiency.

a) Formative assessment and training tools: FIF Platform with AI-Based Scoring.

During the intervention period, the AI system of the FIF oral English training platform was utilized to monitor and provide feedback on students' daily oral practice assignments. The platform primarily functions as an automated assessment tool, evaluating key linguistic aspects including pronunciation, fluency, and vocabulary usage.

b) Summative assessment and criterion tools: Expert blind review

To evaluate the intervention effect of the “Praise-Speak” program on students, this study employed the IELTS Speaking Test assessment criteria (see Appendix C) to conduct pre and post intervention assessments of the speaking performance of the participants (*n* = 13). All speaking assessments were conducted and audio-recorded on the FIF speaking training platform employed in this study. The recordings were subsequently evaluated through a double-blind scoring process by two raters who hold TEM-8 certification. The assessment framework encompassed four dimensions: fluency and coherence, lexical resource, grammatical range and accuracy, and pronunciation. For each dimension, the average score awarded by the two raters was calculated and adopted as the student's final speaking score. To assess inter-rater reliability, the Intraclass Correlation Coefficient (ICC) was computed using the SPSSAU platform. Based on a two-way mixed-effects model with absolute agreement, the ICC value was found to be 0.886 (95% *CI*: 0.620–0.965). According to the benchmark proposed by Koo and Li (2016), an ICC exceeding 0.75 indicates excellent inter-rater consistency, thereby supporting the high reliability of the speaking scores used in this study. This integrated assessment approach enhances the objectivity and credibility of the results through methodological triangulation.

#### 3.3.4 Reflective journal

The reflection log outline for this study was developed in accordance with the first level (Reaction level) of the Kirkpatrick model (Kirkpatrick, 1998; Cahapay, 2021; Evangelista et al., 2025; Jekel et al., 2025; Puripunyavanich, 2025), and was primarily utilized to gather trainees' experiences and perceptions regarding the program (see Appendix D).

### 3.4 Intervention procedure

The intervention process of this study is divided into: pre-test, a 16-week “Praise-Speak” intervention, post-test, and qualitative data collection. Please refer to Figure 3. To ensure the fidelity of the intervention, before the intervention, we provided standardized training to the two instructors on the principles of AE education and the use of the FIF platform. Weekly teaching discussion meetings were held to monitor the consistency of implementation, and students' attendance was recorded throughout the intervention period.

Pre-test: All 13 participants completed the IELTS Speaking Test on FIF to establish their baseline speaking proficiency before intervention. Concurrently, their levels of speaking anxiety were assessed using the FLSAQ. Notably, the anxiety scale data were drawn directly from participants' responses in a prior institution-wide survey (see Section 3.2), minimizing participant fatigue associated with repeated testing and enhancing data consistency.

Intervention: Subsequently, a 16-week “Praise-Speak” program was implemented on a weekly basis, with each session lasting 90 min, once a week. The core components of the program included oral presentation activities, English word chain games, peer and instructor evaluation sessions, and structured confidence-building exercises. The foundational principle of the project was to enable teachers to transform students' efforts and achievements into academically meaningful interpretations and value-based empowerment through professional, constructive verbal feedback. Specifically, this approach involved recognizing students' initial arguments as emerging critical thinking, affirming their attempts to use new vocabulary as measurable progress, and collaboratively providing targeted linguistic tools as scaffolding to help students clearly identify the developmental pathway from “good” to “better,” thereby fostering intrinsic motivation for learning.

Post-test: After the intervention ended, the English proficiency and speaking anxiety levels of all participants were measured again to understand the changes.

For a systematic description of the “Praise-Speak” program core components and operational principles, see Appendix G.

Qualitative data collection: Following the completion of the post-test, all participants were administered written interviews to gather in-depth insights regarding their learning experiences, perceived learning outcomes, and the aspects of the intervention program they found most impactful or challenging. The interview protocol included the following questions, and more details to refer to Appendix D:

- Did you acquire any new knowledge and/or skills from the “Praise-Speak” program?
- Has the “Praise-Speak” program been useful for improving your current studies?
- Do you like this program?

### 3.5 Data analysis methods

Quantitative data were analyzed using SPSS software (Version 26.0). Paired-sample *t*-tests were conducted to assess differences between pre-test and post-test scores. Effect sizes (Cohen's *d*) were calculated for paired-sample *t*-tests to evaluate the practical significance of the findings. For qualitative data specifically reflective logs thematic analysis, as outlined by Braun and Clarke (2006), was employed to facilitate systematic coding and theme identification. To enhance the credibility and reliability of the coding process, two researchers independently performed initial coding of all reflective logs and subsequently resolved discrepancies through collaborative discussion to achieve thematic consensus.

## 4 Results of quantitative research

### 4.1 Demographic characteristics of the sample population

**Figure 4:**
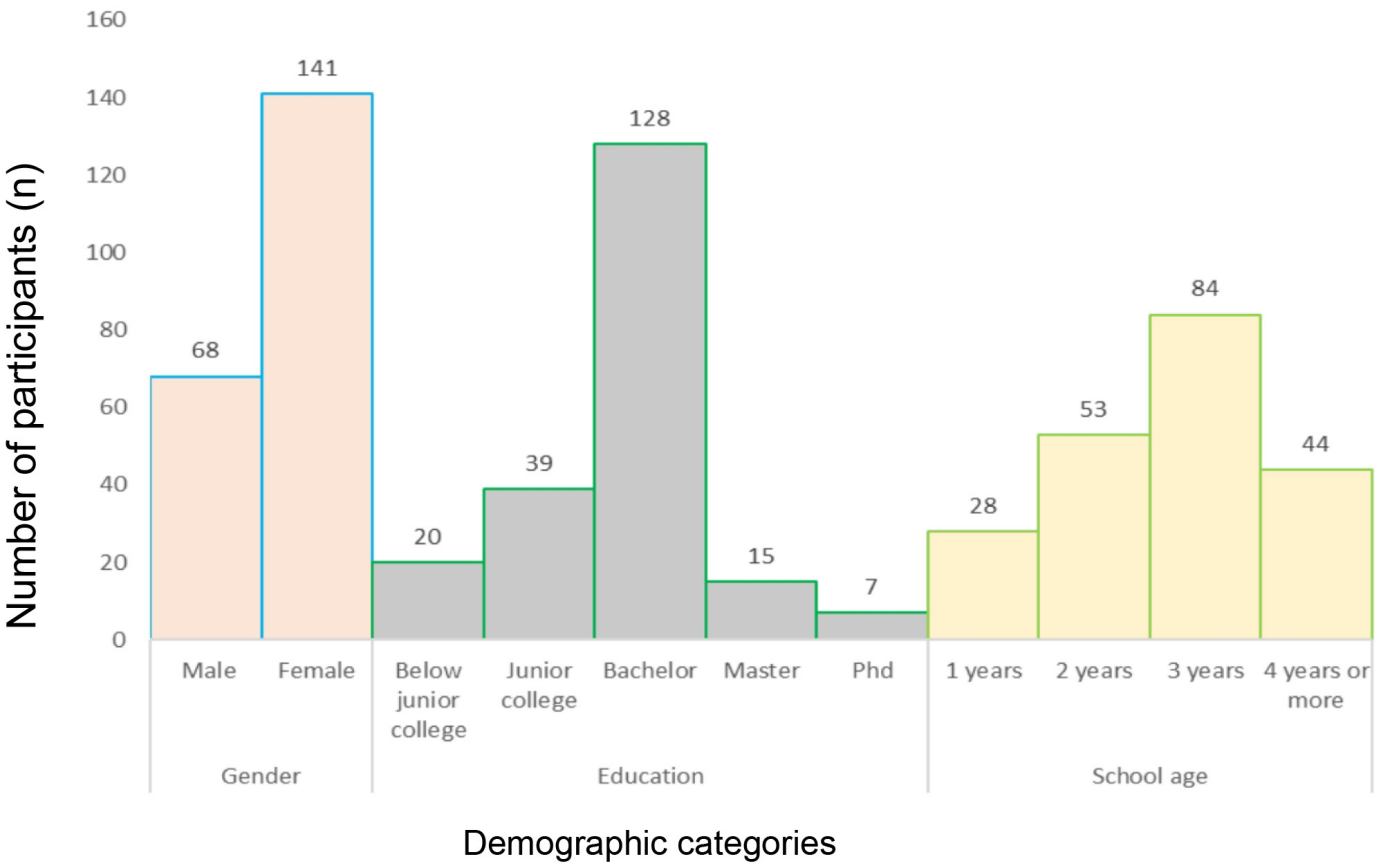
The descriptive statistics for AEDS.

A total of 209 students participated in the Appreciative Education Dimension Scale (AEDS) study. As illustrated in Figure 4, the majority of participants were female (67.46%), undergraduate students (61.24%), and had more than 3 years of English learning experience (61.24%). Notably, although undergraduates comprised the largest proportion of the sample, however, after conducting cross-analysis on the AEDS data of different school ages, it was found that freshmen exhibited the greatest need for appreciative education. These findings serve as a basis for identifying the target population for intervention in this study.

### 4.2 Significant disparities in demand across school ages

Based on the school ages data derived from demographic characteristics, a one-way ANOVA was conducted to examine the demand for appreciative education. The results revealed statistically significant differences among students of different school ages across all five dimensions of the ADES (*p* < 0.05). Importantly, a clear developmental trend was observed: the perceived level of appreciative education needs increased progressively with school age. As presented in Table 1, students with lower school ages consistently exhibited lower mean scores across all dimensions. Notably, first-year students (defined as freshmen in this study) demonstrated the lowest mean scores on each dimension, respectively, The attitude of *AE* = 3.009; Emotional basis = 2.793; Classroom application = 2.884; Student feedback = 2.929; External environment = 2.875.

**Table 1:** The results of the variance analysis for ADES.

Dimension	School age (*M* ±*S*)				*f*	*p*
	1 year (*n* = 28)	2 years (*n* = 53)	3 years (*n* = 84)	4 years or more (*n* = 44)		
The attitude of AE	3.009 ± 1.031	3.132 ± 0.883	3.443 ± 0.562	3.534 ± 0.582	4.890	0.003^**^
Emotional basis	2.793 ± 1.084	3.121 ± 0.885	3.493 ± 0.590	3.568 ± 0.494	9.227	0.000^**^
Classroom application	2.884 ± 1.033	2.925 ± 0.876	3.381 ± 0.597	3.335 ± 0.810	5.588	0.001^**^
Student feedback	2.929 ± 0.772	3.038 ± 0.668	3.238 ± 0.543	3.313 ± 0.579	3.346	0.020^*^
External environment	2.875 ± 1.102	3.099 ± 0.797	3.330 ± 0.621	3.494 ± 0.562	5.095	0.002^**^

*M*, mean; *SD*, standard deviation;

^*^p < 0.05,

^**^p < 0.01.

### 4.3 “Praise-Speak” program effect analysis based on paired samples *t*-test

Given the small sample size (*n* = 13), the normality of the difference scores was assessed using the Shapiro–Wilk test prior to the paired *t*-test. As shown in Table 2, the speaking score differences deviated significantly from normality (*W* = 0.65, *p* < 0.001), while the anxiety differences were normally distributed (*W* = 0.92, *p* = 0.28). As the speaking scores violated the normality assumption, a Wilcoxon signed-rank test was further conducted on the pre- and post-intervention speaking scores as a sensitivity analysis. The results confirmed a significant improvement (*Z* = 3.18, *p* = 0.001).

**Table 2:** Shapiro–Wilk test (on difference scores) and Wilcoxon Signed-rank test (on raw pre/post scores) results.

Variables	Shapiro–Wilk (difference normality)		Wilcoxon signed-rank	
	*W*	*p*	*Z*	*p*
Speaking scores	0.65	0.00	3.18	0.001
Speaking anxiety	0.92	0.28	–	–

Wilcoxon signed-rank tests were performed on the raw pre- and post-intervention scores; Shapiro–Wilk tests were performed on the difference scores (post–pre). The Wilcoxon test was not conducted for anxiety scores as the differences were normally distributed (*p* > 0.05).

*Z* = standardized test statistic; *p* = asymptotic Sig. (2-sided test).

## 5 Qualitative research results

This study utilized thematic analysis (Braun and Clarke, 2006) to further examine students' subjective experiences of the “Praise-Speak” program. Upon completion of the program, reflective logs were collected in accordance with the Kirkpatrick Level 1 (Reaction) model (see Appendix D), and subsequently subjected to coding. The analytical process rigorously adhered to the six phases of thematic analysis:

- Organize and analyze the contents of the students' journals;
- Generate initial codes (e.g., “Learning self-confidence has been enhanced.”, “Dare to try and express.”, “Teachers' encouragement is the driving force for learning.”).
- Summarize the sub-themes and code the theoretical dimensions accordingly.
- The rigor and credibility of the analysis results were ensured through multiple rounds of systematic review and refinement of coding and themes conducted by the researchers.
- The final determination of the matters has been completed.
- The report is presented in Table 2.

## 6 Discussion

### 6.1 The relationship between school ages and demand for appreciative education

RQ1: To what extent do students of different age groups exhibit varying needs for appreciative education, and consequently, which student group demonstrates the most urgent need for intervention through the “Praise-Speak” program?

The results of the variance analysis presented in Table 1 reveal a statistically significant increase in students' perceived levels of appreciative education with age (*p* < 0.05), this trend is further supported by the factor analysis results which consistently indicate lower scores among freshmen across all five dimensions. The Factor analysis combined with ADES can reveal the following findings more effectively (refer to Appendix F, Table F2):

First, there is relatively limited recognition and understanding of AE among students (*A*1 = 0.799, *A*4 = 0.742).

Second, students report lower levels of emotional support stemming from teachers' care, equitable treatment, and the overall classroom learning environment (*B*1 = 0.820, *B*3 = 0.798, *B*5 = 0.746).

Third, they perceive that AE strategies are infrequently applied by teachers in instructional practice (*A*3 = 0.744, *C*2 = 0.846, *C*3 = 0.838).

Furthermore, students exhibit limited confidence in the positive academic outcomes associated with AE (*D*1 = 0.806, *D*2 = 0.802, *D*3 = 0.796).

Finally, freshmen indicate that the institution provides insufficient promotion and institutional support for AE initiatives (*E*1 = 0.884, *E*2 = 0.813, *E*3 = 0.797, *E*4 = 0.796).

In summary, freshmen exhibit the lowest level of AE education and should therefore be prioritized as the primary target group for intervention initiatives. This cohort faces challenges related to both academic competence and a sense of belonging, which can be effectively explained through the lens of SDT (Deci and Ryan, 2012).

Chickering and Reisser (1993) identity development theory can further explain this phenomenon (Chickering and Reisser, 1993). The theory posits that college students' growth experiences are characterized by seven psychological and social development dimensions: Developing Competence, Managing Emotions, Moving through Autonomy toward Interdependence, Developing Mature Interpersonal Relationships, Establishing Identity, Developing Purpose, and Developing Integrity. Freshmen, upon entering a new learning environment and lacking academic accumulation, are in the stage of developing competence and managing emotions, making them more prone to anxiety and self-doubt. It is precisely this heightened sensitivity that accounts for the significantly higher demand for appreciation education among freshmen in this study. Positive feedback, as an external affirmation, helps freshmen build a sense of competence and enhance emotional stability in the early stages of their academic journey. In contrast, upperclassmen have adapted to the learning environment and are less dependent on external affirmation. Combining this developmental perspective with SDT enriches our understanding of how psychological and social development stages moderate students' acceptance of educational interventions. The “Praise-speak” program addresses these dual needs by fostering competence through the FIF platform and enhancing relatedness via AE, thereby providing targeted theoretical and practical interventions.

### 6.2 The analysis of the effectiveness and underlying mechanisms of the “Praise-Speak” program

RQ2: Can the “Praise-Speak” program significantly enhance students' oral performance and substantially reduce their speaking anxiety?

As shown in Table 3, the “Praise-Speak” program significantly improved students' IELTS speaking scores (*p* = 0.005) and extremely statistically significant reduced their levels of speaking anxiety (*p* < 0.001).

**Table 3:** Results of paired samples *t*-test.

Paired variables	Pre-test *M* (*SD*)	Post-test *M* (*SD*)	Average difference	95% *CI*	*t*	*d*f	*p*	Cohen's *d*
Speaking level	5.08 (0.86)	5.69 (0.63)	0.61	[0.22, 1.00]	−3.411	12	0.005	0.95
Anxiety level	5.84 (1.17)	1.72 (0.68)	4.12	[3.25, 4.98]	10.411	12	0.000	2.89

*M*, mean; *SD*, standard deviation; for speaking level, difference = post-test – pre-test; Cohen's *d* interpretation: 0.2 = small, 0.5 = medium, 0.8 = large.

First, the comparison between pre and post intervention speaking scores indicated that students' mean score after the intervention (*M* = 5.69, *SD* = 0.63) was significantly higher than the pre-intervention mean (*M* = 5.08, *SD* = 0.86), reflecting an average improvement of 0.61 points. This difference was statistically significant, *t*(12) = −3.411, *p* = 0.005, the corresponding effect sizes (Cohen's *d*) were 0.95 for speaking scores and 2.89 for anxiety, indicating large and very large practical effects, respectively. It suggesting that the “Praise-Speak” program effectively enhances students' IELTS speaking performance.

Second, students' reported anxiety levels decreased substantially from pre- to post-intervention, with mean scores dropping from 5.84 (*SD* = 1.17) to 1.72 (*SD* = 0.68), representing an average reduction of 4.12. This change was highly statistically significant, *t*(12) = 10.411, *p* < 0.001, providing strong evidence that the Praise Speak program is effective in alleviating foreign language speaking anxiety.

In conclusion, the quantitative findings demonstrate that the “Praise-Speak” program significantly improves students' speaking proficiency (*p* < 0.01) and substantially reduces their speaking anxiety (*p* < 0.001).

While RQ2 quantitatively examined changes in speaking anxiety and oral proficiency, the qualitative analysis revealed motivation as a key explanatory mechanism underlying these outcomes. As shown in Table 4, students' reflective journals consistently pointed to motivational shifts as a driving force behind their improved performance and reduced anxiety. However, given the small sample size (*n* = 13), these findings are preliminary and require replication in larger-scale studies before firm conclusions can be drawn.

**Table 4:** Analysis of themes in student experience (*N* = 13).

Theoretical dimension	Sub-theme (total responses)	Core statement (frequency, *n*)
Learning motivation	Enhancement of self-confidence and sense of efficacy (total responses = 22)	Learning self-confidence has been enhanced/I can do it (10)
		Dare to try and express (5)
		Willing to employ complex expressions (2)
	Establishment of psychological safety (total responses= 25)	It's okay to make mistakes./Don't be afraid of making mistakes (12)
		The learning atmosphere is relaxed and safe (8)
		A positive and inclusive classroom atmosphere (5)
Teaching method	Positive acceptance of appreciative education (total responses= 30)	Teachers' encouragement is the driving force for learning (16)
		Positive feedback can brings a sense of achievement (9)
		The relationship between teachers and students is harmonious (5)
Technology integration	High recognition of the FIF platform (total responses = 25)	FIF offers self-study paths (10)
		The platform is powerful and convenient, and it can record progress (9)
		Instant practice and feedback system (6)
	The synergy between technology and humanism (total responses= 20)	FIF platform and appreciative education are perfectly combined (8)
		The model addresses both skill and psychological barriers at the same time (7)
		The ideal modern learning model (5)
	Suggestions for future improvement (total responses = 6)	Interactive features should be added, such as community interaction or two-person dialogue (3)
		It is suspected that the scores on the FIF platform are not accurate enough (1)
		AI cannot surpass real human teachers (1)
		Insufficient personalized attention (1)

The thematic analysis of the reflection logs (refer to Table 4) suggests that the success of the “Praise-Speak” program can be attributed to three core mechanisms:

a) Learning motivation

Students frequently mentioned “Learning self-confidence has been enhanced/I can do it” (*n* = 10), “Dare to try and express” (*n* = 5), as well as “Willing to employ complex expressions” (*n* = 2). This indicates that the students' self-confidence and sense of efficacy have been enhanced.

Students frequently mentioned “It's okay to make mistakes/Don't be afraid of making mistakes” (*n* = 12), “The learning atmosphere is relaxed and safe” (*n* = 8), “A positive and inclusive classroom atmosphere” (*n* = 5), This finding clearly indicates that fostering students' intrinsic motivation has contributed to enhanced oral performance, while the establishment of psychological safety provides a direct explanation for the significant reduction in speaking anxiety observed in the quantitative data.

b) Learning method

Students regularly mentioned “Teachers' encouragement is the driving force for learning” (*n* = 16), “Positive feedback can bring a sense of achievement” (*n* = 9), “The relationship between teachers and students is harmonious” (*n* = 5), the positive feedback regarding appreciative education was mentioned 30 times, it was the highest frequency across all dimensions. This suggests that AE is well-received among students and plays a significant role in enhancing oral performance and reducing anxiety, as reflected in the quantitative findings.

c) Technology integration

“FIF and appreciative education are perfectly combined” (*n* = 8). This clarifies “The model addresses both skill and psychological barriers at the same time” (*n* = 7), and exemplified by students that “Praise-Speak” program was “The ideal modern learning model” (*n* = 5). This indicates that the co-development of technology and humanistic care is a teaching model favored by students.

The blended teaching model proposed in this study embodies an innovative mechanism of the synergy between technology and humanity. Firstly, the appreciation from teachers serves as an emotional scaffold (Liu et al., 2024), and positive affirmation can reduce the anxiety of EFL learners, thereby enhancing their learning motivation and achieving better teaching outcomes. Secondly, the interaction between students and the FIF platform enhances their sense of social presence creating a sense of connection and support (Sprott et al., 2025; Harati et al., 2025; Deci and Ryan, 2012), transforming isolated practice into a socially meaningful experience, which is precisely the manifestation of “relatedness” emphasized in SDT. Under the combined effect of these two mechanisms, the “Prise-Speak” learning process forms a virtuous cycle, thus making this blended teaching a self-sustaining learning ecosystem.

Research findings suggest that the integration of AE with technology-assisted teaching models has yielded significant outcomes in reducing speaking anxiety and improving both oral proficiency and learning motivation. This effect may be attributed to the FIF platform's capacity to deliver immediate and objective feedback, functioning as an effective tool for enhancing students' language abilities. At the same time, appreciative education addresses the lack of humanistic support often associated with technological tools by reinforcing learners' psychological motivation. Consequently, the synergistic combination of AE and digital platforms demonstrates a notable impact on enhancing oral performance, with learning motivation serving as a key mediating mechanism.

Recent scholarly investigations into artificial intelligence enhanced language learning have underscored the critical role of addressing learners' affective needs (Ma et al., 2025; Shi, 2026; Shuai, 2025), and the “Praise-Speak” program constitutes a targeted pedagogical response to this imperative. This observed synergy is highly consistent with the latest research findings in the field. Fan and Zhang (2024) emphasized the critical importance of considering psychological factors, especially anxiety, in artificial intelligence-assisted language learning environments. This study expands on this research direction by providing empirical evidence that a humanistic appreciative education approach can directly alleviate such anxiety. This blended teaching method is conducive to unlocking the full potential of AI tools like the FIF platform, which Fan and Zhang (2024) argue depends on learners' positive emotional investment and attitude toward technology. Therefore, the collaborative integration of AE with digital platforms not only stimulates English learning motivation and oral performance but also compensates for the key deficiencies in purely technology-driven models. The aforementioned analysis provides robust support and triangulation for addressing RQ2 of this study (Tashakkori and Teddlie, 2010; Creswell and Clark, 2017).

Both quantitative and qualitative research demonstrate that the program of this research not only generates positive outcomes in the short term, but also fosters key components essential for the sustainable development of language learning. The program integrated with the FIF platform and combined with teachers' offline teaching based appreciative education, it strengthened students' learning motivation, and improved their speaking English performance. It effectively addresses key challenges in the EFL learning process, particularly low self-efficacy and negative emotional experiences associated with oral expression. The “Praise-Speak” program initiative not only supports students in building psychological safety and strengthening intrinsic motivation but also cultivates a sustainable psychological resource that enhances their adaptability to future academic and personal challenges, aligning with the fundamental principles of sustainable development education. Furthermore, this study offers higher education researchers an innovative, scalable, and sustainable pedagogical model that promotes learner motivation and contributes to the realization of United Nations Sustainable Development Goal 4: ensuring inclusive and equitable quality education.

## 7 Limitation

Firstly, with regard to the measurement instruments, this study primarily employed exploratory factor analysis (EFA) and reliability indices to assess the structural configuration and internal consistency of the two core scales (FLSAQ and AEDS). However, confirmatory factor analysis (CFA) or structural equation modeling (SEM) was not utilized to further validate the construct validity of these scales in independent samples. While this limitation does not compromise the internal coherence of the study's findings, it suggests that the generalizability of the scale structures may be constrained. Furthermore, it is worth noting that although the one-dimensional structure of FLSAQ is reasonable in psychometrics, it may not fully capture the multifaceted nature of speaking anxiety (e.g., cognitive anxiety, physiological anxiety). Future research should aim to corroborate these results using more rigorous psychometric methods to enhance the robustness and cross-contextual applicability of the measurement models.

Secondly, with regard to research design, the intervention effect observed in this study is derived from a single-group pre-test–post-test pilot study involving a limited sample size (*n* = 13) drawn from a single institution. As a feasibility study, this research lacks a control group, which compromises internal validity and limits causal inference. Specifically, threats such as history, maturation, regression to the mean, testing effects, expectancy effects, and selection bias cannot be ruled out. Additionally, the sample is drawn exclusively from a single geographic site, thereby constraining the external validity and generalizability of the findings. Future studies should employ a randomized controlled trial (RCT) design to evaluate the effectiveness of this model in larger, more diverse samples, and to examine its applicability across different cultural contexts and levels of language proficiency.

Thirdly, while all participants expressed uniformly positive evaluations of the “Praise-Speak” program, and no contradictory or dissenting views emerged during the interviews. However, it might also be due to self-selection bias or social desirability bias in the interview environment. To enhance validity, future studies should intentionally recruit a more demographically and experientially diverse participant pool and adopt methodological safeguards against bias, such as anonymous self-report surveys or interviews conducted by independent, non-affiliated researchers.

Fourthly, with regard to deepening the intervention, the emotional support component in this study was primarily delivered through teachers to dominated offline appreciative education. Given the variability across teachers and educational institutions (Kavanoz and Yuksel, 2017), the current approach faces challenges in scalability and consistent implementation. To address this limitation, a promising direction for future research is to operationalize the core principles of appreciative education, such as positive affirmation and strength-based focus into intelligent, algorithm-driven feedback systems on digital platforms. For instance, developing an AI-powered platform capable of providing real-time, encouraging feedback could enable artificial intelligence to serve as a scalable source of emotional support, fostering learners' sense of competence and relatedness. Therefore, the key to achieving large scale and sustainable application of this teaching model, which is full of humanistic care, lies in how to free it from the reliance on specific teachers.

Fifthly, concerning the underlying mechanism of action, although this study demonstrated the significant efficacy of the integrated model in reducing anxiety, it did not examine the specific mediating roles of perceived competence and relatedness. Future studies should incorporate more rigorous mediation analysis models to uncover the psychological pathways through which these factors exert their influence. Therefore, future research should aim to elucidate the psychological mechanisms underlying its functioning. A clearer understanding of these processes would enable college educators to refine their instructional designs and implement targeted teaching interventions that more effectively promote learners' mental health while simultaneously enhancing their academic competencies.

## 8 Conclusion

In terms of **Theoretical contributions**, this study integrates key theoretical frameworks from educational psychology specifically, Self-Determination Theory (SDT) and the Zone of Proximal Development (ZPD) into a coherent operational model, offering a novel theoretical lens through which to understand emotional intervention in technology enhanced learning environments.

In terms of **Practical Implications**, this study proposes a replicable and data basic teaching model termed “Praise-Speak”, fostering the synergistic integration of technological application and humanistic care to support students' lifelong development.

In terms of **Sustainable Education**, the “Prise-Speak” program in this study not only produced positive learning outcomes but also cultivated learners' psychological construction and self-efficacy, which can continuously support their lifelong learning outside the classroom. Moreover, the “technology-humanism synergy” can achieve inclusive and equitable quality education, which is highly consistent with the principles of Sustainable Development Goal 4 (SDG 4) in the field of education.

This research gradually explores a new research direction, that is, to what extent future artificial intelligence systems can simulate appreciative feedback to provide scalable emotional support. Transforming the core concepts of appreciation education into algorithm-driven feedback mechanisms is expected to enable teaching models focused on humanistic care to break away from the reliance on specific teachers and promote the large-scale and sustainable application of emotionally supportive language education.

## Data Availability

The datasets presented in this study can be found in online repositories. The names of the repository/repositories and accession number(s) can be found in the article/Supplementary material.
