# Supplementary material for: Integrating appreciative education with AI-assisted oral training for sustainable EFL learning: a study on speaking anxiety and oral proficiency
Source: Front Psychol. 2026 Apr 10;17:1803848. doi: 10.3389/fpsyg.2026.1803848 (PMC13106310; doi:10.3389/fpsyg.2026.1803848)
Supplement: Supplementary file 1 [file Data_Sheet_1.pdf]

Appendix A. Foreign Language Speaking Anxiety Questionnaire

This questionnaire was designed to gather information about your English Language Speaking Anxiety level in the classroom setting. After reading each statement, please circle the number that best reffects your opinion. There were no correct or incorrect answers to any of the items in the questionnaire. Thank you for your comments.

'1' Strongly disagree (SD), '2' Disagree (D), '3' Not sure (NS), '4' Agree (A), '5' Strongly agree (SA)

Table A1. Foreign Language Speaking Anxiety Questionnaire Items.

Statements	SD	D	NS	A	SA
I am never quite sure of myself when I am speaking in English.					
I am afraid of making mistakes in English classes.					
I tremble when I know that I am going to be called on in English classes.					
I get frightened when I don't understand what the teacher is saying in English.					
I start to panic when I have to speak without preparation in English classes.					
I get embarrassed to volunteer answers in English classes.					
I feel nervous while speaking English with native speakers.					
I get upset when I don't understand what the teacher is correcting.					
I don't feel conffdent when I speak English in classes.					
I am afraid that my English teacher is ready to correct every mistake I make.					
I can feel my heart pounding when I am going to be called on in English classes.					
I always feel that the other students speak English better than I do.					
I feel very self-conscious about speaking English in front of other students.					
I get nervous and confused when I am speaking in English classes.					
I get nervous when I don't understand every word my English teacher says.					
I feel overwhelmed by the number of rules I have to learn to speak English.					
I am afraid the other students will laugh at me when I speak English.					
I get nervous when the English teacher asks questions which I haven't prepared in advance.					
