# Supplementary material for: Integrating appreciative education with AI-assisted oral training for sustainable EFL learning: a study on speaking anxiety and oral proficiency
Source: Front Psychol. 2026 Apr 10;17:1803848. doi: 10.3389/fpsyg.2026.1803848 (PMC13106310; doi:10.3389/fpsyg.2026.1803848)
Supplement: Supplementary file 2 [file Data_Sheet_2.pdf]

Appendix B. Appreciative Education Dimension Scale

This questionnaire aims to understand your perceptions and expectations regarding Appreciative Education (AE) in routine teaching. Your responses are anonymous and there are no right or wrong answers. Thank you for your participation.

Part 1: Demographic Information

(Please tick [√] the option that applies to you)

1. Your gender is：

A. Boy B. Girl

2. Your education background is：

A. Junior college and below B. junior college C. undergraduate D. Master E. PHD

3. How many years have you been in school：

A. One year B. Two years C. Three years D. Four years

Part 2: Appreciative Education Attitudes and Practices

The following section contains statements about your views and experiences. Please indicate your level of agreement with each statement by ticking [√] the corresponding number (1-5).

‘1’ Strongly disagree (SD), ‘2’ Disagree (D), ‘3’ Not sure (NS), ‘4’ Agree (A), ‘5’ Strongly agree (SA)

Table B1. Appreciative Education Dimension Scale Items.

	Statements	SD	D	NS	A	SA
The attitude of AE	A1. Do you consider it essential to incorporate appreciation education into the interest-based instruction of this course?					
	A2. You frequently engage in discussions about appreciation education with your classmates.					
	A3. How frequently do teachers implement appreciation-based education in classroom settings?					
	A4. You have a good understanding of appreciation-based education.					
Emotional Basis	B1. Do you think teachers can treat all students equally?					
	B2. You really like this major.					
	B3. You think that the teachers at school care deeply about the students.					
	B4. You are already very passionate about this course.					
	B5. Your relationship with most of the teachers and students is very harmonious.					
Classroom Applicati	C1. Do you think that compared with praising students privately, teachers' public praise can be more effective?					

on	C2. Do you think the teacher has actively encouraged you to participate in activities and, based on this, provided you with more learning support and encouragement?
	C3. Do you think the teacher frequently uses positive language to praise you during the teaching process?
	C4. In your opinion, compared with students who perform well academically, students who have difficulty in learning need more praise.
Student Feedback	D1. You believe that appreciation education has had a positive impact on an individual's learning outcomes.
	D2. You think that in the subjects involved in appreciation education, there will be obvious improvement in exam results.
	D3. You are likely to develop a greater appreciation for this subject due to the teacher's consistent affirmation and support experienced during class.
	D4. You recognize that the teacher is employing the approach of appreciative education.
External Environ ment	E1. Do you believe the school is promoting the implementation of appreciation education in the classroom through formal policies or other structured initiatives?
	E2. It is believed that schools should encourage students to actively participate in club activities, promote positive psychological traits, and, on this basis, support teachers in implementing appreciation-based education approaches in the classroom.
	E3. Do you believe that educational institutions frequently organize lectures or training sessions pertaining to appreciation education?
	E4. Your school strongly promotes an appreciation-based educational approach.
