# Supplementary material for: Integrating appreciative education with AI-assisted oral training for sustainable EFL learning: a study on speaking anxiety and oral proficiency
Source: Front Psychol. 2026 Apr 10;17:1803848. doi: 10.3389/fpsyg.2026.1803848 (PMC13106310; doi:10.3389/fpsyg.2026.1803848)
Supplement: Supplementary file 3 [file Data_Sheet_3.pdf]

Appendix C. IELTS Speaking Test Rubric

Table C1. IELTS Speaking Test Scoring Rubric.

Criterion	1	2	3	4	5	6	7	8	9	10	11	12	13
Fluency													
Vocabulary													
Grammar													
Pronunciation													
Score													

Appendix D. Reflection Journal Prompts (Kirkpatrick Level 1)

1. Did you acquire any new knowledge and/or skills from the Praise Speak program?
2. Has the Praise Speak program been useful for improving your current studies?
3. Do you like this program?
4. Do you think the Praise Speak program has an inspiring effect on your oral expression?
5. What's your evaluation of the training instructor and the Fit platform for this learning program?
6. Do you think the classroom atmosphere of Praise Speak is positive?
7. Does the training method combining the FIF platform with appreciative education meet your learning needs?
8. Overall, do you think this is a high-quality training program?
