# Supplementary material for: Integrating appreciative education with AI-assisted oral training for sustainable EFL learning: a study on speaking anxiety and oral proficiency
Source: Front Psychol. 2026 Apr 10;17:1803848. doi: 10.3389/fpsyg.2026.1803848 (PMC13106310; doi:10.3389/fpsyg.2026.1803848)
Supplement: Supplementary file 4 [file Data_Sheet_4.pdf]

Appendix E. Foreign Language Speaking Anxiety Questionnaire Reliability and validity analysis

Table E1. Adaptability test of FLSAQ.

KMO and Bartlett's Test		
Kaiser-Meyer-Olkin Measure of Sampling Adequacy.		0.976
Bartlett's Test of Sphericity	Approx. Chi-Square	14227.302
	df	153
	Sig.	0.000

Table E2. The Exploratory Factor Analysis results of FLSAQ.

Factors Derived from the Exploratory Factor Analysis		
Items	Initial	Extraction
1. I am never quite sure of myself when I am speaking in English.	1.000	.521
2. I am afraid of making mistakes in English classes.	1.000	.607
3. I tremble when I know that I am going to be called on in English classes.	1.000	.624
4. I get frightened when I don’t understand what the teacher is saying in English.	1.000	.581
5. I start to panic when I have to speak without preparation in English classes.	1.000	.654
6. I get embarrassed to volunteer answers in English classes.	1.000	.564
7. I feel n1ervous while speaking English with native speakers.	1.000	.519
8. I get upset when I don’t understand what the teacher is correcting.	1.000	.554
9. I don’t feel confident when I speak English in classes.	1.000	.708
10. I am afraid that my English teacher is ready to correct every mistake I make.	1.000	.534
11. I can feel my heart pounding when I am going to be called on in English classes.	1.000	.627
12. I always feel that the other students speak English better than I do.	1.000	.580
13. I feel very self-conscious about speaking English in front of other students.	1.000	.676
14. I get nervous and confused when I am speaking in English classes.	1.000	.746
15. I get nervous when I don’t understand every word my English teacher says.	1.000	.646
16. I feel overwhelmed by the number of rules I have to learn to speak English.	1.000	.570
17. I am afraid the other students will laugh at me when I speak English.	1.000	.601
18. I get nervous when the English teacher asks questions which I haven’t prepared in advance.	1.000	.635

Values express loadings

Table E3. Explanation of total variance.

Component	Initial Eigenvalues			Extraction Sums of Squared Loadings		
	Total	% of Variance	Cumulative %	Total	% of Variance	Cumulative %
1	10.947	60.817	60.817	10.947	60.817	60.817
2	.807	4.483	65.300			
3	.671	3.725	69.025			
4	.603	3.349	72.374			
5	.529	2.939	75.313			
6	.496	2.754	78.067			
7	.454	2.523	80.591			
8	.423	2.349	82.940			
9	.412	2.287	85.227			
10	.381	2.116	87.343			
11	.353	1.963	89.306			
12	.341	1.894	91.200			
13	.323	1.797	92.997			
14	.282	1.568	94.564			
15	.273	1.516	96.081			
16	.255	1.418	97.499			
17	.242	1.343	98.842			
18	.209	1.158	100.000			

Extraction Method: Principal Component Analysis.

Figure E1. Scree Plot of FLSAQ.

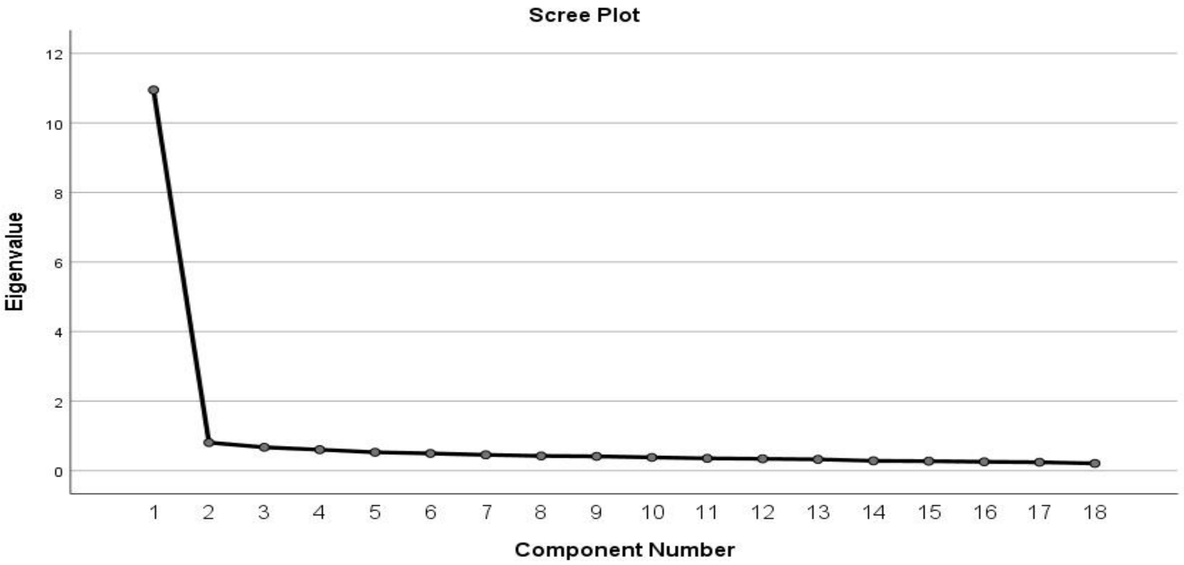

Table E4. Reliability statistics for the FLSAQ.

Scale Section	Items	Cronbach's Alpha
Overall Scale	Q1-Q18	0.962
Part 1	Q1-Q9	0.925
Part 2	Q10-Q18	0.934

*N = (1044). The Spearman-Brown coefficient of the total scale is 0.948.
