# Supplementary material for: Integrating appreciative education with AI-assisted oral training for sustainable EFL learning: a study on speaking anxiety and oral proficiency
Source: Front Psychol. 2026 Apr 10;17:1803848. doi: 10.3389/fpsyg.2026.1803848 (PMC13106310; doi:10.3389/fpsyg.2026.1803848)
Supplement: Supplementary file 5 [file Data_Sheet_5.pdf]

Appendix F. Appreciative Education Dimension Scale Reliability and Validity Analysis

Table F1. Adaptability test of AEDS.

Dimension	Kaiser-Meyer-Olkin Measure of Sampling Adequacy.	Bartlett's Test of Sphericity		
		Approx. Chi-Square	df	Sig.
The attitude of AE	0.704	220.253	6	.000
Emotional Basis	0.748	475.168	10	.000
Classroom Application	0.802	326.390	6	.000
Student Feedback	0.762	269.732	6	.000
External Environment	0.705	407.062	6	.000

Table F2. The Exploratory Factor Analysis results of AEDS.

Factors Derived from the Exploratory Factor Analysis		
Dimension	Items text	Component
The attitude of AE	A1. Do you consider it essential to incorporate appreciation education into the interest-based instruction of this course?	.799
	A2. You frequently engage in discussions about appreciation education with your classmates.	.785
	A3. How frequently do teachers implement appreciation-based education in classroom settings?	.744
	A4. You have a good understanding of appreciation-based education.	.742
Emotional Basis	B1. Do you think teachers can treat all students equally?	.820
	B2. You really like this major.	.806
	B3. You think that the teachers at school care deeply about the students.	.798
	B4. You are already very passionate about this course.	.783
	B5. Your relationship with most of the teachers and students is very harmonious.	.746
Classroom Application	C1. Do you think that compared with praising students privately, teachers' public praise can be more effective?	.856
	C2. Do you think the teacher has actively encouraged you to participate in activities and, based on this, provided you with more learning support and encouragement?	.846
	C3. Do you think the teacher frequently uses positive language to praise you during the teaching process?	.838
	C4. In your opinion, compared with students who perform well academically, students who have difficulty in learning need more praise.	.735
Student	D1. You believe that appreciation education has had a	.806

Feedback	positive impact on an individual's learning outcomes.	
	D2. You think that in the subjects involved in appreciation education, there will be obvious improvement in exam results.	.802
	D3. You are likely to develop a greater appreciation for this subject due to the teacher's consistent affirmation and support experienced during class.	.796
	D4. You recognize that the teacher is employing the approach of appreciative education.	.789
External Environment	E1. Do you believe the school is promoting the implementation of appreciation education in the classroom through formal policies or other structured initiatives?	.884
	E2. It is believed that schools should encourage students to actively participate in club activities, promote positive psychological traits, and, on this basis, support teachers in implementing appreciation-based education approaches in the classroom.	.813
	E3. Do you believe that educational institutions frequently organize lectures or training sessions pertaining to appreciation education?	.797
	E4. Your school strongly promotes an appreciation-based educational approach.	.796

Values express loadings.

Table F3. Total Variance Explained for the AEDS.

Dimension	Component	Initial Eigenvalues			Extraction Sums of Squared Loadings		
		Total	% of Variance	Cumulative %	Total	% of Variance	Cumulative %
The attitude of AE	1	2.358	58.952	58.952	2.358	58.952	58.952
	2	.739	18.487	77.439			
	3	.534	13.358	90.797			
	4	.368	9.203	100.000			
Emotional Basis	1	3.129	62.571	62.571	3.129	62.571	62.571
	2	.759	15.179	77.751			
	3	.512	10.231	87.982			
	4	.394	7.879	95.861			
	5	.207	4.139	100.000			
Classroom Application	1	2.691	67.285	67.285	2.691	67.285	67.285

	2	.590	14.756	82.042			
	3	.368	9.204	91.246			
	4	.350	8.754	100.000			
Student Feedback	1	2.550	63.748	63.748	2.550	63.748	63.748
	2	.601	15.034	78.782			
	3	.489	12.228	91.010			
	4	.360	8.990	100.000			
External Environment	1	2.712	67.793	67.793	2.712	67.793	67.793
	2	.745	18.617	86.410			
	3	.345	8.621	95.031			
	4	.199	4.969	100.000			

Extraction method: Principal Component Analysis.

Table F4. Reliability statistics for the AEDS.

Scale Section	Cronbach's Alpha
The attitude of AE	0.766
Emotional Basis	0.85
Classroom Application	0.833
Student Feedback	0.810
External Environment	0.841
