# Supplementary material for: Integrating appreciative education with AI-assisted oral training for sustainable EFL learning: a study on speaking anxiety and oral proficiency
Source: Front Psychol. 2026 Apr 10;17:1803848. doi: 10.3389/fpsyg.2026.1803848 (PMC13106310; doi:10.3389/fpsyg.2026.1803848)
Supplement: Supplementary file 6 [file Data_Sheet_6.pdf]

Appendix G. A systematic description of the“Praise-Speak” program

1. The theoretical basis

The "Praise-Speak" project is a blended teaching model that combines offline appreciation education with AI-assisted oral English training based on the FIF platform. This model is grounded in Vygotsky's "Zone of Proximal Development" theory and Deci and Ryan's "Self-Determination Theory". By simultaneously fulfilling learners' cognitive and emotional needs, it aims to enhance oral proficiency and reduce oral anxiety.“

2. Core components

a) Appreciation Education Module: This module is implemented in face-to-face offline classrooms. Teachers offer feedback based on students' strengths, transforming students' efforts and achievements into academically meaningful recognition. For instance, they view students' initial arguments as "the sprouts of critical thinking" and affirm their attempts to use new vocabulary as "quantifiable progress". They provide targeted language support, assist students in planning better development paths, and offer more verbal praise as well as written commendations in the review of assignments.

b) AI-assisted module: The FIF Oral English Training Platform offers structured oral tasks and provides immediate feedback on pronunciation, fluency, and vocabulary usage.

3. The core active

Online weekly scheduled oral presentation assignment practice；
An English word chain game in class；
The peer review and teacher comment sections based on the principle of appreciation education；
Structured self-confidence building exercises.

4. Implementing principle

Through collaborative scaffolding teaching, teachers help students clearly identify the development path for improving their oral language skills, thereby fostering their intrinsic learning motivation and classroom psychological safety. The class is held once a week for 90 minutes, lasting for 16 weeks. The class follows a stable process: warm-up activities (word games), student oral reports, appreciative evaluation, and confidence-building activities.
